# 1,3-Dicyclo­hexyl-1-(4-nitro­benzo­yl)urea

**DOI:** 10.1107/S1600536810016107

**Published:** 2010-05-08

**Authors:** A. N. Dhinaa, R. Jagan, K. Sivakumar, K. Chinnakali

**Affiliations:** a Department of Physics, Anna University Chennai, Chennai 600 025, India

## Abstract

In the title compound, C_20_H_27_N_3_O_4_, both cyclo­hexane rings adopt chair conformations. The benzene ring and the amide group are oriented at a dihedral angle of 62.1 (2)°. In the crystal structure, inter­molecular N—H⋯O and C—H⋯O hydrogen bonds link the mol­ecules into chains propagating in [010], which contain *R*_2_^2^(12) ring motifs.

## Related literature

For the biological activity of benzoyl­urea and *N*-aroylurea derivatives, see: Song *et al.* (2008 ▶, 2009 ▶); Amornraksa *et al.* (2009 ▶). For related *N*-benzoyl-*N*,*N*′-dicyclo­hexyl­urea structures, see: Orea Flores *et al.* (2006 ▶); Wang & Peng (2008 ▶).
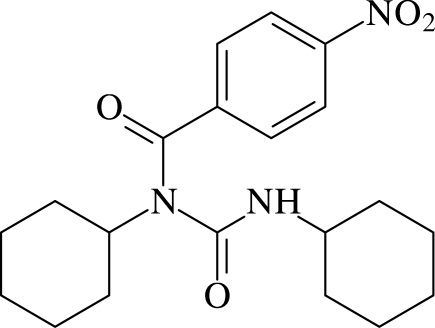


## Experimental

### 

#### Crystal data

C_20_H_27_N_3_O_4_
*M* *_r_* = 373.45
Monoclinic, 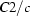

*a* = 25.294 (2) Å
*b* = 9.5757 (7) Å
*c* = 16.6943 (14) Å
β = 105.140 (2)°
*V* = 3903.1 (5) Å^3^
*Z* = 8
Mo *K*α radiation
μ = 0.09 mm^−1^
*T* = 292 K
0.25 × 0.20 × 0.20 mm

#### Data collection

Bruker Kappa APEXII CCD diffractometer
Absorption correction: multi-scan (*SADABS*; Bruker 1999 ▶) *T*_min_ = 0.836, *T*_max_ = 0.982
22623 measured reflections
5012 independent reflections
3152 reflections with *I* > 2σ(*I*)
*R*_int_ = 0.031

#### Refinement

*R*[*F* ^2^ > 2σ(*F* ^2^)] = 0.049
*wR*(*F* ^2^) = 0.138
*S* = 1.02
5012 reflections
244 parameters
H-atom parameters constrained
Δρ_max_ = 0.15 e Å^−3^
Δρ_min_ = −0.14 e Å^−3^

### 

Data collection: *APEX2* (Bruker, 2004 ▶); cell refinement: *SAINT-Plus* (Bruker, 2004 ▶); data reduction: *SAINT-Plus*; program(s) used to solve structure: *SHELXS97* (Sheldrick, 2008 ▶); program(s) used to refine structure: *SHELXL97* (Sheldrick, 2008 ▶); molecular graphics: *ORTEP-3* (Farrugia, 1997 ▶) and *Mercury* (Macrae *et al.*, 2008 ▶); software used to prepare material for publication: *PLATON* (Spek, 2009 ▶).

## Supplementary Material

Crystal structure: contains datablocks I, global. DOI: 10.1107/S1600536810016107/hb5432sup1.cif

Structure factors: contains datablocks I. DOI: 10.1107/S1600536810016107/hb5432Isup2.hkl

Additional supplementary materials:  crystallographic information; 3D view; checkCIF report

## Figures and Tables

**Table 1:** Hydrogen-bond geometry (Å, °)

*D*—H⋯*A*	*D*—H	H⋯*A*	*D*⋯*A*	*D*—H⋯*A*
N1—H1*A*⋯O1^i^	0.86	2.10	2.9396 (15)	166
C1—H1⋯O2^ii^	0.98	2.43	3.3636 (18)	160

Symmetry codes: (i) 
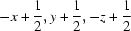
; (ii) 
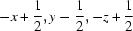
.

**Fig. 1.**
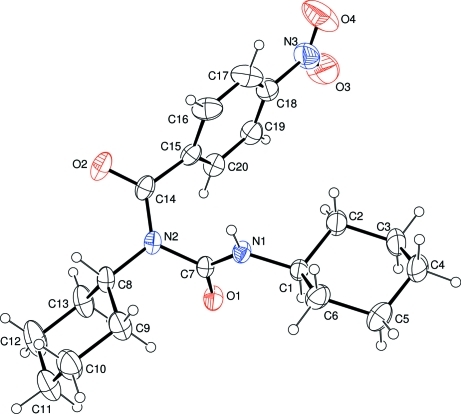
The molecular structure of (I) with displacement ellipsoids drawn at the 30% probability level.

**Fig. 2.**
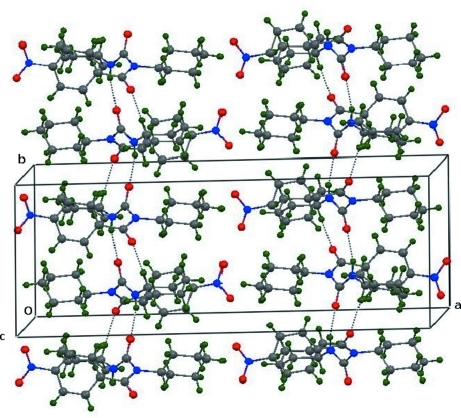
Part of the crystal structure of (I), showing fused R22(12) ring motifs generating a molecular ribbon extending along the b axis.

## supplementary crystallographic information

### Comment

Benzoylurea derivatives act as antimitotic (Song *et al.*, 2008) and antiproliferative (Song *et al.*, 2009) agents. Some of the N-aroylurea analogs have been found to exhibit antioxidant activity (Amornraksa *et al.*, 2009). We report here the crystal structure of the title compound, (I), an aroylurea.

In the title molecule (Fig. 1), both cyclohexane rings adopt chair conformations. The benzoyl carbonyl group is twisted away from the N–H group and as a result no intramolecular N—H···O hydrogen bond is formed. The dihedral angle between the N1/C7/O1 and N2/C14/O2 planes is 65.0 (2)°. The amide group (C7/O1/N1) and the benzene ring (C15–C20) are oriented at a dihedral angle of 62.1 (1)°. The nitro group is almost coplanar with the attached benzene ring [C19—C18—N3—O3 = -0.8 (3)° and C17—C18—N3—O4 = -1.6 (3)°]. Bond lengths and angles are comparable to those observed in *N*-benzoyl-*N*,*N*'-dicyclohexylurea (Orea Flores *et al.*, 2006) and *N*-(4-bromobenzoyl)-*N*,*N*-dicyclohexylurea (Wang & Peng, 2008).

In the crystal structure, N1—H1A···O1^i^ and C1—H1···O2^ii^ hydrogen bonds (symmetry codes as in Table 1) generate R^2^_2_(12) ring motifs which are fused into a ribbon-like structure along the *b* axis (Fig.2).

### Experimental

4-Nitrobenzoic acid (1 g, 5.9 mmol) and dicyclohexylcarbodiimide (1.203 g, 5.9 mmol) were dissolved in dichloromethane (30 ml). The resulting mixture was stirred overnight and then the solvent was removed by rotary evaporator. The product was isolated by column chromatography by using ethyl acetate-hexane (1:9) as eluent. Pale yellow blocks of (I) were obtained by slow evaporation of an ethanolic solution over a period of two weeks.

### Refinement

H atoms were initially located in a difference Fourier map and later placed in idealized positions and constrained to ride on their parent atoms, with N–H = 0.86 Å, C–H = 0.93–0.98 Å and *U*_iso_(H) = 1.2*U*_eq_(C,*N*). The U^ij^ parameters of the nitro group atoms indicate possible disorder but attempts to model the disorder were not successful. Beamstop affected reflection 200 was omitted during the refinement.

### Crystal data

**Table:**

C_20_H_27_N_3_O_4_	*F*(000) = 1600
*M**_r_* = 373.45	*D*_x_ = 1.271 Mg m^−^^3^
Monoclinic, *C*2/*c*	Mo *K*α radiation, λ = 0.71073 Å
Hall symbol: -C 2yc	Cell parameters from 4827 reflections
*a* = 25.294 (2) Å	θ = 2.5–23.4°
*b* = 9.5757 (7) Å	µ = 0.09 mm^−^^1^
*c* = 16.6943 (14) Å	*T* = 292 K
β = 105.140 (2)°	Block, pale-yellow
*V* = 3903.1 (5) Å^3^	0.25 × 0.20 × 0.20 mm
*Z* = 8	

### Data collection

**Table:**

Bruker Kappa APEXII CCD diffractometer	5012 independent reflections
Radiation source: fine-focus sealed tube	3152 reflections with *I* > 2σ(*I*)
graphite	*R*_int_ = 0.031
ω and φ scan	θ_max_ = 28.7°, θ_min_ = 2.3°
Absorption correction: multi-scan (*SADABS*; Bruker 1999)	*h* = −34→34
*T*_min_ = 0.836, *T*_max_ = 0.982	*k* = −12→12
22623 measured reflections	*l* = −22→22

### Refinement

**Table:**

Refinement on *F*^2^	Primary atom site location: structure-invariant direct methods
Least-squares matrix: full	Secondary atom site location: difference Fourier map
*R*[*F*^2^ > 2σ(*F*^2^)] = 0.049	Hydrogen site location: inferred from neighbouring sites
*wR*(*F*^2^) = 0.138	H-atom parameters constrained
*S* = 1.02	*w* = 1/[σ^2^(*F*_o_^2^) + (0.0549*P*)^2^ + 1.482*P*] where *P* = (*F*_o_^2^ + 2*F*_c_^2^)/3
5012 reflections	(Δ/σ)_max_ = 0.001
244 parameters	Δρ_max_ = 0.15 e Å^−^^3^
0 restraints	Δρ_min_ = −0.14 e Å^−^^3^

### Special details

**Table:**

Geometry. All esds (except the esd in the dihedral angle between two l.s. planes) are estimated using the full covariance matrix. The cell esds are taken into account individually in the estimation of esds in distances, angles and torsion angles; correlations between esds in cell parameters are only used when they are defined by crystal symmetry. An approximate (isotropic) treatment of cell esds is used for estimating esds involving l.s. planes.
Refinement. Refinement of *F*^2^ against ALL reflections. The weighted *R*-factor wR and goodness of fit *S* are based on *F*^2^, conventional *R*-factors *R* are based on *F*, with *F* set to zero for negative *F*^2^. The threshold expression of *F*^2^ > σ(*F*^2^) is used only for calculating *R*-factors(gt) etc. and is not relevant to the choice of reflections for refinement. *R*-factors based on *F*^2^ are statistically about twice as large as those based on *F*, and *R*- factors based on ALL data will be even larger.

### Fractional atomic coordinates and isotropic or equivalent isotropic displacement parameters (Å^2^)

**Table:**

	*x*	*y*	*z*	*U*_iso_*/*U*_eq_	
C1	0.18614 (6)	0.20858 (14)	0.12793 (9)	0.0419 (3)	
H1	0.1969	0.1116	0.1220	0.050*	
C2	0.12619 (7)	0.2094 (2)	0.12851 (11)	0.0651 (5)	
H2A	0.1151	0.3039	0.1374	0.078*	
H2B	0.1212	0.1517	0.1738	0.078*	
C3	0.09045 (7)	0.1538 (3)	0.04623 (12)	0.0795 (6)	
H3A	0.0991	0.0563	0.0402	0.095*	
H3B	0.0522	0.1596	0.0466	0.095*	
C4	0.09915 (8)	0.2346 (2)	−0.02572 (12)	0.0786 (6)	
H4A	0.0870	0.3301	−0.0228	0.094*	
H4B	0.0775	0.1938	−0.0770	0.094*	
C5	0.15886 (8)	0.2338 (2)	−0.02557 (11)	0.0713 (5)	
H5A	0.1638	0.2902	−0.0713	0.086*	
H5B	0.1700	0.1391	−0.0337	0.086*	
C6	0.19480 (8)	0.29061 (19)	0.05559 (10)	0.0603 (4)	
H6A	0.2330	0.2850	0.0549	0.072*	
H6B	0.1860	0.3880	0.0615	0.072*	
C7	0.25502 (6)	0.18506 (14)	0.26110 (9)	0.0405 (3)	
C8	0.34370 (6)	0.27308 (16)	0.35126 (10)	0.0511 (4)	
H8	0.3545	0.3551	0.3869	0.061*	
C9	0.36104 (7)	0.2997 (2)	0.27233 (12)	0.0665 (5)	
H9A	0.3436	0.3841	0.2460	0.080*	
H9B	0.3492	0.2226	0.2341	0.080*	
C10	0.42312 (8)	0.3153 (3)	0.29111 (15)	0.0843 (6)	
H10A	0.4335	0.3269	0.2396	0.101*	
H10B	0.4345	0.3982	0.3246	0.101*	
C11	0.45178 (8)	0.1898 (3)	0.33654 (17)	0.0955 (8)	
H11A	0.4911	0.2037	0.3493	0.115*	
H11B	0.4427	0.1081	0.3013	0.115*	
C12	0.43525 (8)	0.1653 (3)	0.41589 (16)	0.1003 (8)	
H12A	0.4471	0.2434	0.4532	0.120*	
H12B	0.4531	0.0817	0.4428	0.120*	
C13	0.37307 (7)	0.1487 (2)	0.39846 (13)	0.0748 (6)	
H13A	0.3617	0.0644	0.3663	0.090*	
H13B	0.3631	0.1393	0.4505	0.090*	
C14	0.25633 (7)	0.32416 (15)	0.38509 (9)	0.0478 (4)	
C15	0.19665 (6)	0.29435 (15)	0.37139 (9)	0.0462 (3)	
C16	0.16036 (8)	0.40464 (17)	0.36337 (13)	0.0689 (5)	
H16	0.1732	0.4956	0.3632	0.083*	
C17	0.10558 (9)	0.3818 (2)	0.35558 (14)	0.0784 (6)	
H17	0.0811	0.4560	0.3489	0.094*	
C18	0.08794 (7)	0.2471 (2)	0.35787 (11)	0.0611 (4)	
C19	0.12263 (7)	0.13583 (19)	0.36631 (11)	0.0599 (4)	
H19	0.1095	0.0453	0.3674	0.072*	
C20	0.17750 (7)	0.15920 (16)	0.37322 (10)	0.0521 (4)	
H20	0.2016	0.0842	0.3791	0.063*	
N1	0.22096 (5)	0.26334 (11)	0.20560 (7)	0.0426 (3)	
H1A	0.2192	0.3510	0.2157	0.051*	
N2	0.28337 (5)	0.25964 (12)	0.33451 (7)	0.0450 (3)	
N3	0.02944 (8)	0.2204 (3)	0.35113 (12)	0.0897 (6)	
O1	0.26427 (4)	0.06149 (10)	0.25260 (6)	0.0535 (3)	
O2	0.27950 (5)	0.40101 (12)	0.44180 (7)	0.0660 (3)	
O3	0.01453 (7)	0.1016 (3)	0.35244 (15)	0.1366 (8)	
O4	−0.00053 (8)	0.3209 (3)	0.34562 (14)	0.1356 (8)	

### Atomic displacement parameters (Å^2^)

**Table:**

	*U*^11^	*U*^22^	*U*^33^	*U*^12^	*U*^13^	*U*^23^
C1	0.0420 (8)	0.0411 (7)	0.0381 (8)	0.0019 (6)	0.0026 (6)	−0.0032 (6)
C2	0.0507 (10)	0.0968 (13)	0.0464 (10)	−0.0144 (9)	0.0101 (8)	−0.0049 (9)
C3	0.0459 (10)	0.1293 (18)	0.0568 (12)	−0.0117 (11)	0.0016 (8)	−0.0154 (12)
C4	0.0766 (14)	0.0941 (14)	0.0491 (11)	0.0318 (11)	−0.0122 (9)	−0.0059 (10)
C5	0.0924 (15)	0.0818 (12)	0.0369 (9)	−0.0024 (10)	0.0122 (9)	0.0032 (8)
C6	0.0683 (11)	0.0700 (10)	0.0455 (9)	−0.0077 (9)	0.0199 (8)	−0.0026 (8)
C7	0.0382 (7)	0.0415 (7)	0.0391 (8)	−0.0064 (6)	0.0052 (6)	−0.0011 (6)
C8	0.0437 (8)	0.0566 (8)	0.0464 (9)	−0.0144 (7)	0.0000 (7)	−0.0014 (7)
C9	0.0496 (10)	0.0845 (12)	0.0617 (11)	−0.0081 (9)	0.0079 (8)	0.0146 (9)
C10	0.0558 (12)	0.1142 (17)	0.0825 (15)	−0.0205 (11)	0.0175 (10)	0.0039 (13)
C11	0.0434 (11)	0.1230 (19)	0.109 (2)	−0.0024 (12)	−0.0003 (11)	−0.0069 (16)
C12	0.0559 (12)	0.1229 (19)	0.1000 (19)	−0.0031 (12)	−0.0191 (12)	0.0269 (15)
C13	0.0529 (11)	0.0874 (13)	0.0695 (13)	−0.0088 (9)	−0.0099 (9)	0.0259 (10)
C14	0.0610 (10)	0.0460 (7)	0.0338 (8)	−0.0122 (7)	0.0075 (7)	0.0021 (6)
C15	0.0602 (10)	0.0465 (8)	0.0329 (7)	−0.0064 (7)	0.0142 (7)	−0.0015 (6)
C16	0.0856 (14)	0.0472 (9)	0.0866 (14)	0.0010 (8)	0.0453 (11)	0.0004 (9)
C17	0.0818 (14)	0.0768 (13)	0.0902 (15)	0.0217 (10)	0.0466 (12)	0.0135 (11)
C18	0.0551 (10)	0.0856 (12)	0.0461 (10)	−0.0019 (9)	0.0198 (8)	0.0029 (8)
C19	0.0637 (11)	0.0619 (10)	0.0558 (10)	−0.0155 (8)	0.0188 (8)	−0.0026 (8)
C20	0.0582 (10)	0.0475 (8)	0.0505 (9)	−0.0055 (7)	0.0140 (7)	0.0030 (7)
N1	0.0465 (7)	0.0358 (6)	0.0405 (7)	0.0001 (5)	0.0020 (5)	−0.0033 (5)
N2	0.0433 (7)	0.0482 (7)	0.0384 (7)	−0.0094 (5)	0.0019 (5)	−0.0027 (5)
N3	0.0614 (12)	0.1421 (19)	0.0727 (12)	−0.0012 (13)	0.0299 (9)	0.0005 (12)
O1	0.0563 (7)	0.0400 (5)	0.0544 (7)	0.0028 (4)	−0.0028 (5)	−0.0017 (5)
O2	0.0836 (9)	0.0709 (7)	0.0413 (6)	−0.0298 (6)	0.0121 (6)	−0.0137 (5)
O3	0.0837 (12)	0.1591 (19)	0.183 (2)	−0.0499 (13)	0.0636 (13)	−0.0461 (16)
O4	0.0759 (11)	0.192 (2)	0.1528 (19)	0.0377 (13)	0.0546 (12)	0.0442 (16)

### Geometric parameters (Å, °)

**Table:**

C1—N1	1.4613 (17)	C10—C11	1.503 (3)
C1—C6	1.504 (2)	C10—H10A	0.97
C1—C2	1.519 (2)	C10—H10B	0.97
C1—H1	0.98	C11—C12	1.508 (4)
C2—C3	1.530 (2)	C11—H11A	0.97
C2—H2A	0.97	C11—H11B	0.97
C2—H2B	0.97	C12—C13	1.531 (3)
C3—C4	1.493 (3)	C12—H12A	0.97
C3—H3A	0.97	C12—H12B	0.97
C3—H3B	0.97	C13—H13A	0.97
C4—C5	1.510 (3)	C13—H13B	0.97
C4—H4A	0.97	C14—O2	1.2213 (17)
C4—H4B	0.97	C14—N2	1.365 (2)
C5—C6	1.521 (2)	C14—C15	1.494 (2)
C5—H5A	0.97	C15—C16	1.383 (2)
C5—H5B	0.97	C15—C20	1.385 (2)
C6—H6A	0.97	C16—C17	1.375 (3)
C6—H6B	0.97	C16—H16	0.93
C7—O1	1.2217 (16)	C17—C18	1.369 (3)
C7—N1	1.3213 (18)	C17—H17	0.93
C7—N2	1.4377 (17)	C18—C19	1.364 (3)
C8—N2	1.4834 (19)	C18—N3	1.477 (3)
C8—C13	1.512 (2)	C19—C20	1.381 (2)
C8—C9	1.515 (2)	C19—H19	0.93
C8—H8	0.98	C20—H20	0.93
C9—C10	1.526 (2)	N1—H1A	0.86
C9—H9A	0.97	N3—O3	1.200 (3)
C9—H9B	0.97	N3—O4	1.213 (3)
			
N1—C1—C6	110.17 (12)	C9—C10—H10A	109.4
N1—C1—C2	111.33 (12)	C11—C10—H10B	109.4
C6—C1—C2	110.94 (13)	C9—C10—H10B	109.4
N1—C1—H1	108.1	H10A—C10—H10B	108.0
C6—C1—H1	108.1	C10—C11—C12	111.1 (2)
C2—C1—H1	108.1	C10—C11—H11A	109.4
C1—C2—C3	110.45 (15)	C12—C11—H11A	109.4
C1—C2—H2A	109.6	C10—C11—H11B	109.4
C3—C2—H2A	109.6	C12—C11—H11B	109.4
C1—C2—H2B	109.6	H11A—C11—H11B	108.0
C3—C2—H2B	109.6	C11—C12—C13	110.85 (17)
H2A—C2—H2B	108.1	C11—C12—H12A	109.5
C4—C3—C2	111.43 (17)	C13—C12—H12A	109.5
C4—C3—H3A	109.3	C11—C12—H12B	109.5
C2—C3—H3A	109.3	C13—C12—H12B	109.5
C4—C3—H3B	109.3	H12A—C12—H12B	108.1
C2—C3—H3B	109.3	C8—C13—C12	110.96 (16)
H3A—C3—H3B	108.0	C8—C13—H13A	109.4
C3—C4—C5	110.71 (15)	C12—C13—H13A	109.4
C3—C4—H4A	109.5	C8—C13—H13B	109.4
C5—C4—H4A	109.5	C12—C13—H13B	109.4
C3—C4—H4B	109.5	H13A—C13—H13B	108.0
C5—C4—H4B	109.5	O2—C14—N2	122.40 (15)
H4A—C4—H4B	108.1	O2—C14—C15	119.65 (15)
C4—C5—C6	111.38 (16)	N2—C14—C15	117.92 (12)
C4—C5—H5A	109.4	C16—C15—C20	119.26 (16)
C6—C5—H5A	109.4	C16—C15—C14	119.19 (14)
C4—C5—H5B	109.4	C20—C15—C14	121.35 (14)
C6—C5—H5B	109.4	C17—C16—C15	120.97 (16)
H5A—C5—H5B	108.0	C17—C16—H16	119.5
C1—C6—C5	110.49 (14)	C15—C16—H16	119.5
C1—C6—H6A	109.6	C18—C17—C16	118.38 (17)
C5—C6—H6A	109.6	C18—C17—H17	120.8
C1—C6—H6B	109.6	C16—C17—H17	120.8
C5—C6—H6B	109.6	C19—C18—C17	122.23 (17)
H6A—C6—H6B	108.1	C19—C18—N3	118.54 (18)
O1—C7—N1	125.28 (13)	C17—C18—N3	119.23 (19)
O1—C7—N2	120.81 (12)	C18—C19—C20	119.19 (16)
N1—C7—N2	113.87 (12)	C18—C19—H19	120.4
N2—C8—C13	111.79 (13)	C20—C19—H19	120.4
N2—C8—C9	111.58 (12)	C19—C20—C15	119.96 (16)
C13—C8—C9	111.80 (16)	C19—C20—H20	120.0
N2—C8—H8	107.1	C15—C20—H20	120.0
C13—C8—H8	107.1	C7—N1—C1	123.38 (11)
C9—C8—H8	107.1	C7—N1—H1A	118.3
C8—C9—C10	110.73 (15)	C1—N1—H1A	118.3
C8—C9—H9A	109.5	C14—N2—C7	122.22 (12)
C10—C9—H9A	109.5	C14—N2—C8	120.06 (12)
C8—C9—H9B	109.5	C7—N2—C8	117.59 (12)
C10—C9—H9B	109.5	O3—N3—O4	124.0 (2)
H9A—C9—H9B	108.1	O3—N3—C18	118.4 (2)
C11—C10—C9	111.10 (17)	O4—N3—C18	117.5 (2)
C11—C10—H10A	109.4		
			
N1—C1—C2—C3	179.16 (15)	C17—C18—C19—C20	0.3 (3)
C6—C1—C2—C3	56.1 (2)	N3—C18—C19—C20	−179.59 (16)
C1—C2—C3—C4	−56.0 (2)	C18—C19—C20—C15	−0.1 (3)
C2—C3—C4—C5	56.1 (2)	C16—C15—C20—C19	0.6 (2)
C3—C4—C5—C6	−56.5 (2)	C14—C15—C20—C19	175.33 (14)
N1—C1—C6—C5	179.75 (13)	O1—C7—N1—C1	4.9 (2)
C2—C1—C6—C5	−56.51 (19)	N2—C7—N1—C1	−177.33 (12)
C4—C5—C6—C1	56.7 (2)	C6—C1—N1—C7	−126.35 (15)
N2—C8—C9—C10	179.16 (15)	C2—C1—N1—C7	110.13 (16)
C13—C8—C9—C10	−54.8 (2)	O2—C14—N2—C7	−171.22 (13)
C8—C9—C10—C11	55.8 (2)	C15—C14—N2—C7	10.96 (19)
C9—C10—C11—C12	−57.2 (3)	O2—C14—N2—C8	4.6 (2)
C10—C11—C12—C13	56.8 (3)	C15—C14—N2—C8	−173.21 (12)
N2—C8—C13—C12	−179.41 (17)	O1—C7—N2—C14	−121.45 (16)
C9—C8—C13—C12	54.7 (2)	N1—C7—N2—C14	60.66 (18)
C11—C12—C13—C8	−55.3 (3)	O1—C7—N2—C8	62.63 (18)
O2—C14—C15—C16	53.2 (2)	N1—C7—N2—C8	−115.27 (14)
N2—C14—C15—C16	−128.91 (16)	C13—C8—N2—C14	97.56 (18)
O2—C14—C15—C20	−121.55 (17)	C9—C8—N2—C14	−136.42 (15)
N2—C14—C15—C20	56.33 (19)	C13—C8—N2—C7	−86.42 (17)
C20—C15—C16—C17	−1.3 (3)	C9—C8—N2—C7	39.60 (18)
C14—C15—C16—C17	−176.15 (17)	C19—C18—N3—O3	−0.8 (3)
C15—C16—C17—C18	1.5 (3)	C17—C18—N3—O3	179.3 (2)
C16—C17—C18—C19	−1.0 (3)	C19—C18—N3—O4	178.3 (2)
C16—C17—C18—N3	178.90 (17)	C17—C18—N3—O4	−1.6 (3)

### Hydrogen-bond geometry (Å, °)

**Table:**

*D*—H···*A*	*D*—H	H···*A*	*D*···*A*	*D*—H···*A*
N1—H1A···O1^i^	0.86	2.10	2.9396 (15)	166
C1—H1···O2^ii^	0.98	2.43	3.3636 (18)	160

Symmetry codes: (i) −*x*+1/2, *y*+1/2, −*z*+1/2; (ii) −*x*+1/2, *y*−1/2, −*z*+1/2.
